# An Aggregated Mutual Information Based Feature Selection with Machine Learning Methods for Enhancing IoT Botnet Attack Detection

**DOI:** 10.3390/s22010185

**Published:** 2021-12-28

**Authors:** Mohammed Al-Sarem, Faisal Saeed, Eman H. Alkhammash, Norah Saleh Alghamdi

**Affiliations:** 1 College of Computer Science and Engineering, Taibah University, Medina 42353, Saudi Arabia; msarem@taibahu.edu.sa; 2 School of Computing and Digital Technology, Birmingham City University, Birmingham B4 7XG, UK; 3 Department of Computer Science, College of Computers and Information Technology, Taif University, P.O. Box 11099, Taif 21944, Saudi Arabia; Eman.kms@tu.edu.sa; 4 College of Computer and Information Sciences, Princess Nourah Bint Abdulrahman University, Riyadh 11671, Saudi Arabia

**Keywords:** intrusion detection systems, Internet of Things, botnet attack detection, feature selection, machine learning, ensemble methods

## Abstract

Due to the wide availability and usage of connected devices in Internet of Things (IoT) networks, the number of attacks on these networks is continually increasing. A particularly serious and dangerous type of attack in the IoT environment is the botnet attack, where the attackers can control the IoT systems to generate enormous networks of “bot” devices for generating malicious activities. To detect this type of attack, several Intrusion Detection Systems (IDSs) have been proposed for IoT networks based on machine learning and deep learning methods. As the main characteristics of IoT systems include their limited battery power and processor capacity, maximizing the efficiency of intrusion detection systems for IoT networks is still a research challenge. It is important to provide efficient and effective methods that use lower computational time and have high detection rates. This paper proposes an aggregated mutual information-based feature selection approach with machine learning methods to enhance detection of IoT botnet attacks. In this study, the N-BaIoT benchmark dataset was used to detect botnet attack types using real traffic data gathered from nine commercial IoT devices. The dataset includes binary and multi-class classifications. The feature selection method incorporates Mutual Information (MI) technique, Principal Component Analysis (PCA) and ANOVA f-test at finely-granulated detection level to select the relevant features for improving the performance of IoT Botnet classifiers. In the classification step, several ensemble and individual classifiers were used, including Random Forest (RF), XGBoost (XGB), Gaussian Naïve Bayes (GNB), k-Nearest Neighbor (k-NN), Logistic Regression (LR) and Support Vector Machine (SVM). The experimental results showed the efficiency and effectiveness of the proposed approach, which outperformed other techniques using various evaluation metrics.

## 1. Introduction

Internet of Things (IoT) networks are becoming essential components for different advanced applications such as smart cities and smart homes. They provide wide connectivity between the connected devices, with the number of networks growing exponentially every day [1]. The IoT improves the quality of life by providing different types of smart services and applications in several domains, including health care, automation, industrial processes and smart environments [2]. According to Greengard [3], it is predicted that 21.5 billion IoT devices will be used by 2025. This huge number of devices will be vulnerable to different types of attacks that raise several security and privacy issues.

With this rapid development in the internet and its smart connected devices, the number of attacks that affect individuals and businesses has already increased [4]. One of the main applications to improve information security is the use of what are called Intrusion Detection Systems (IDSs), which help to provide a secure environment by identifying and classifying security threats within the internet. Because of the special characteristics of IoT systems, including the dynamics of their networks, and limited battery power and processor capacity, intrusion detection for IoT networks is considered a major challenge, as it needs to consider the trade-off between accuracy of detection and performance overheads [5]. Thus, according to Arshad et al. [5], the main features of IDSs should be: (1) efficient computational and communication overhead, and (2) high detection accuracy.

One of the dangerous threats in IoT networks is what are known as botnets, which can be described as a collection of different bots that are controlled by the Botmaster (behind-the-scenes attacker) using the Command and Control (C&C) channel [6]. The IoT botnet attack works to recruit vulnerable IoT devices in order to generate enormous networks of “bot” devices to generate large numbers of malicious activities that can be controlled remotely by the Botmaster [7]. The attackers can use botnets for stealing data, granting access to devices and performing Distributed Denial-of-Service attacks (DDoS). This attack uses a series of connected devices in order to take down a website or networks for the purpose of disrupting operations in these environments or stopping the main services of the target application [7]. Therefore, detecting and preventing the botnets is very important in computer security and has attracted several researchers to improve the IoT botnet attack detection rate.

Recently, different methods have been proposed and applied to detect IoT botnet attacks. For instance, Popoola et al. [8] proposed a deep learning-based botnet attack detection method to deal with imbalanced traffic data in networks. They utilized a recurrent neural network method for learning hierarchical feature representations of the balanced data to carry out the classification. The authors found that this imbalanced data affected the detection performance, using evaluation measures such as precision, recall and F1 score. The proposed method obtained 99.50%, 99.75% and 99.62% for precision, recall and F1 scores, respectively. In addition, Soe et al. [9] proposed a botnet attack detection method based on Machine Learning (ML) and Sequential Architecture. In this work, the authors adopted a Feature Selection (FS) method to produce a high-performance and lightweight detection system. This system obtained an accuracy of 99% for detecting the botnet attacks using an artificial neural network, J48 decision tree and naïve Bayes. To compare the many machine learning methods that have been applied for botnet attack detection, Tuan et al. [10] conducted experiments for performance evaluation of several machine learning methods for botnet DDoS attack detection using two datasets. The experiments included the use of Support Vector Machine (SVM), Artificial Neural Network (ANN), Naïve Bayes (NB), Decision Tree (DT) and Unsupervised Learning (UL). The outcomes of this research showed that the unsupervised learning methods obtained better detection rates compared to the other machine learning methods.

As the main features of IDSs for IoT networks are the efficiency of the computational and communication overhead and the high detection accuracy [5], the high dimensionality of IoT traffic data affects the efficiency of the detection systems. This paper proposed an aggregated mutual information-based feature selection approach with machine learning methods to enhance the efficiency and performance of IoT botnet attack detection. A freely available benchmark dataset was used to show the benefit of the proposed aggregated feature selection method. Based on an intensively review of the existing available datasets, the N-BaIoT dataset (http://archive.ics.uci.edu/ml/datasets/detection_of_IoT_botnet_attacks_N_BaIoT (last accessed on: 6 December 2021; 23:00 GMT)) was chosen to be used in this research.

The main contributions of this research paper can be summarized as follows:

- The IoT Botnet attack detection is explored as a multiclass classification problem using a dataset with more than 6.2 M instances. The description of the dataset is presented in Section 3.1.
- A feature selection-based method is proposed that incorporates Mutual Information (MI) technique, Principal Component Analysis (PCA) and an ANOVA f-test at finely granulated detection level.
- A fine-granulated aggregated mutual information is proposed and tested on the benchmark dataset. The proposed technique effectively selects the relevant features for increasing the performance of IoT Botnet classifiers.
- A comprehensive and practical approach is proposed that investigates the performance of the proposed technique using two ensemble-based machine learning methods, namely Random Forest (RF) and XGBoost (XGB), and four standalone classifiers, namely, Gaussian Naïve Bayes (GNB), k-Nearest Neighbor (k-NN), Logistic Regression (LR) and Support Vector Machine (SVM).
- Finally, the proposed approach outperforms other techniques using various evaluation metrics.

The rest of the paper is organized as follows: Section 2 reviews the recent studies on IoT botnet attack detection. Section 3 presents the materials and methods used in the present study, while Section 4 highlights and discusses the main results of the proposed approach. Finally, Section 5 concludes the whole paper.

## 2. Related Works

Although the increased usage and growth of information and computer technology makes life easier, it also leads to many security issues as the number of attackers has increased rapidly. One of the important security mechanisms proposed to support information security and protect businesses from dangerous network attacks is known as the intrusion detection system [11]. Several intrusion detection systems based on machine learning and deep learning methods have been proposed for IoT Environments. For instance, Kiran et al. [12] applied NB, SVM, DT and Adaboost methods to detect the attacks (sniffing and poisoning) on IoT networks. They used IoT-based normal and attack data in order to build the model. The applied methods obtained high accuracy rates (0.9895, 0.9895 and 1.00 for SVM, Adaboost and DT respectively). However, these authors indicate that challenges still exist in generating high quality datasets using diverse IoT devices in order to enhance the robustness of the used machine learning models.

Pacheco et al. [13] proposed an artificial neural network-based method for implementing an adaptive IDS to detect attacks on fog nodes in IoT applications and ensure the availability of communication, allowing the nodes to continuously deliver the important information to the end users. The proposed method was able to detect the normal behavior of fog nodes and was able to detect anomalies due to different sources, such as misuses, cyber-attacks, with a high detection rate and low false alarms. In addition, Ferrag et al. [14] proposed an IDS for IoT networks called RDTIDS, which combines REP Tree, JRip algorithm and Random Forest methods. The proposed system used a BoT-IoT dataset and obtained high accuracy in the detection rate compared to the previous studies.

In another study, Amouri et al. [15] proposed an IDS for mobile IoT networks, which involved two stages: (1) Collecting data from dedicated sniffers and generating correctly classified instances that are sent to super node, (2) linear regression performed by the super node to detect the benign and malicious nodes. The proposed system was able to detect the malicious activities (blackhole and DDoS) attacks with detection rates of more than 98% for the high power/node velocity case and 90% for the low power/node velocity case. Similarly, Verma and Ranga [16] used different machine learning methods to detect Denial-of-Service (DoS) attacks on IoT networks. They used different popular datasets and applied statistical methods to evaluate the significant differences between the methods used. They discussed how to select the best classification method based on the application requirements and recommended using ensemble methods to develop IDSs. In addition, Hindy et al. [17] investigated six machine learning methods for an IoT intrusion detection system to detect one type of IoT attack, known as a Message Queuing Telemetry Transport (MQTT) attack. The results showed the effectiveness of the machine learning methods used and emphasized the importance of using flow-based features to detect MQTT-based attacks.

Lv et al. [18] proposed a misuse IDS that depends on specific attack signatures to detect normal and malicious activities, based on an extreme learning machine with a hybrid kernel function. They used the Kernel Principal Component Analysis (KPCA) method for feature selection and feature extraction of the intrusion detection data. The experimental results showed high detection rates and time-saving when using the proposed method. For IoT networks, Gad, Nashat and Barkat [19] used a chi-square feature selection method with different machine learning methods (using binary and multi-class data) on a dataset from a large-scale and diverse IoT network. The experiment showed that the XGBoost classifier outperformed other methods.

Feature selection methods were also used to enhance the detection of IoT botnet attacks. For instance, Alqahtani, Mathkour and Ben Ismail [20] concluded that it is still a challenge to develop an efficient IDS for IoT devices. To address this, they proposed a feature selection method (using a Fisher-score) with a genetic-based XGBoost classifier to obtain a subset of features for detecting IoT botnet attacks. They conducted experiments on a public botnet dataset and it was found that high detection rates were obtained by using only three features. Similarly, Bahşi, Nõmm and La Torre [21] investigated the importance of improved feature selection for reducing the number of features to detect the IoT bots. They showed that a small number of features can obtain high detection rates using a multi-class classifier such as a decision tree. In addition, Panda, Abd Allah and Hassanien [22] developed an efficient feature engineering model with machine learning and deep learning methods for detecting IoT-botnet attacks. To provide efficient detection, two feature engineering methods, K-Medoid sampling and scatter search-based, were applied to obtain optimal feature subsets for the representative dataset. The experimental results showed that the proposed method combined a high detection rate with low computational cost (4.7 s for training and 0.61 s for testing).

Feature selection methods were used in different research disciplines to enhance the proposed machine learning models, for instance IDS for vehicular ad hoc networks [23], drone intrusion detection [24], clickbait detection on social media [25], detection of diseases in health informatics [26] and virtual screening for molecular similarity searching [27]. In addition to machine learning methods for IDS in IoT, several deep learning methods were applied for intrusion detection systems in IoT, which are discussed in [28]. Although there are several studies in the literature addressing the IoT intrusion detection, more research efforts are needed to consider the special characteristics and challenges of IoT systems, which including the limited battery power and processor capacity. According to [5], it is needed to consider the trade-off between accuracy of detection and performance overheads to provide efficient computational and communication overhead, and high detection accuracy. Therefore, this paper proposes a feature selection-based method with several machine learning methods to enhance the performance of IoT Botnet classifiers. The feature selection methods include Mutual Information (MI), Principal Component Analysis (PCA) and ANOVA f-test at fine-granulated detection level.

## 3. Materials and Methods

In this section, the N-BaIoT benchmark dataset is presented and discussed briefly. The data preprocessing and label encoding processes are then explained. Then, the well-known One-versus-the-Rest (OvR) classification technique was used for dealing with multiclass classification problems. Finally, this section describes the methodology used, including details of the choice of classifiers, feature selection methods and the evaluation criteria.

**Figure 1:**
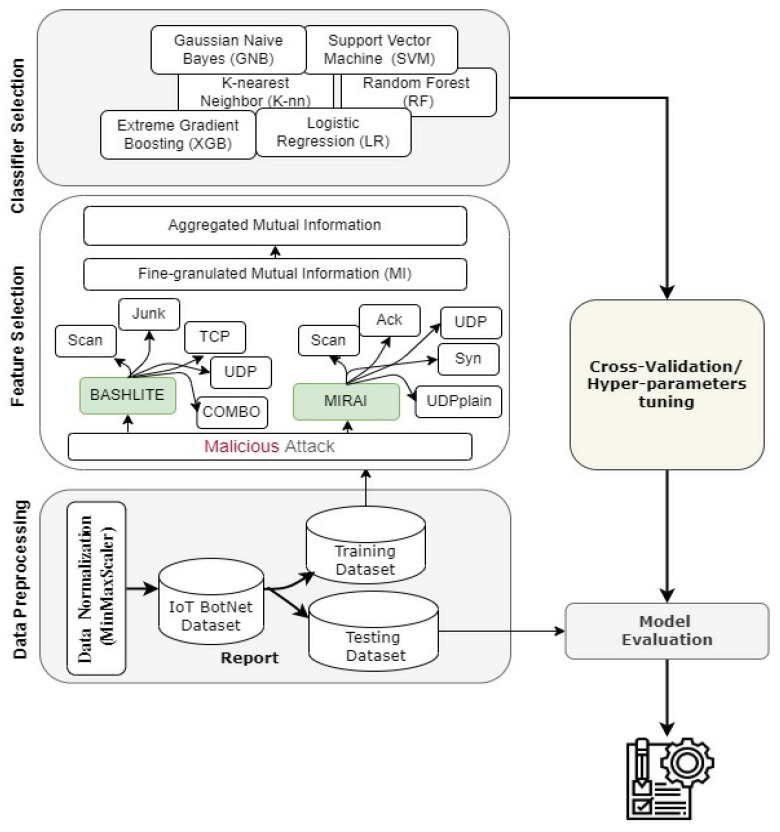
The framework of the proposed approach.

The methodology followed in this research is presented in Figure 1, that includes: data collection, data preparation, feature selection and classifier selection, which is trained and tested on the benchmark dataset with hyper-parameter tuning of the ML models. To evaluate these models, the classifiers were trained and tested without applying any feature selection method. This step helped to measure the efficiency of the used feature selection techniques and investigate their influence on the performance of the ML model. In addition, two data preprocessing techniques were applied: standardization and minimum-maximum normalization (which is known as min–max normalization). Each attack type was then fed into the feature selection methods to obtain a set of reduced features. Subsequently, the set with reduced features was used for training the ML classifiers, using the OvR strategy. The hyper-parameter of the winner ML classifier was then tuned using k-fold cross. In the last phase, the performance of ML classifiers was reported.

### 3.1. Used Dataset

The N-BaIoT data set that is used in this paper is designed to detect botnet attack types, using nine IoT devices that provided the real traffic data [29]. The IoT devices were attacked by two botnet attack families, namely Bashlite and Mirai. In total, there are about five million items of data, grouped in separate files. Each file contains 115 features and a class label. The dataset has also been constructed to server binary classification as well as multi-class classification, where the target class labels take values of “benign” or “TCP attack” for binary classification and “Bashlite” or “Miria” attack types for multi-class classification.

Table 1 below and Table A1 (see Appendix A) show the detailed statistics of the N-BaIoT dataset and the complete list of extracted features. The data records are encoded as L0.01, L0.1, L1, L3 and L5 with respect to the network stream time windows. In addition, the socket and channel category are enriched with additional information about the packet size. For each category, the packet count, mean, packet size and variance are calculated From Table 1. it is obvious that the dataset is organized in a way that allows both binary classification and multi-class classification to be addressed. In this study, as mentioned earlier, the multi-class classification will be investigated, where the number of instances for benign and different attack subclass types is presented in Table 2.

As the distribution of data records is obviously not balanced, the pseudocode presented in Algorithm 1 was used to sample the instances of “Bashlite” attack types and “Mirai” attack types.
**Algorithm 1** Pseudocode of Dataset Sampling**Input**: A list of N-BaIoT files F**Output**: Balanced dataset
DF← an empty lists← size of data frame**for each** file f∈F
**do**: Import the file f as data frame df Count the size $s^{df}$ of the df Append data frame to df∈DF**End for**threshold θ← the smallest data frame size s(df)**For all**df∈DF AND s(df)>θ
**do**: p← percent of data % Sample dataset as $df_{i}\leftarrow \left(\frac{\theta }{df}\right)*100\%$**End For** Return $df_{i}$ as csv format**End**

### 3.2. Data Preprocessing

Although data preprocessing is tedious and time consuming [30,31], its necessity is proven not only for simplifying the machine learning training process but also for improving the effectiveness of the overall processes. Consequently, this study proposes the following pre-preprocessing steps: label encoding, min–max normalization and standardization.

#### 3.2.1. Label Encoding

As the class label contains 11 different categorical values (including one “Benign” class and 10 attack type subclasses), it is not acceptable to feed these values directly to the ML classifiers. Therefore, these features are encoded into numerical values before using the models. In the literature, there are several approaches for encoding the categorical values: one-hot encoding [32], ordinal encoding [33], similarity encoding [34], entity embedding [35] and multi-hot encoding [36]. Among of these, the most used approaches are one-hot and ordinal encoding [37]. For encoding the categorical values found in the class label, this study applies the one-hot encoding approach and transforms each categorical value into a vector of binary variables. It should be noted that applying a one-hot encoding approach leads to increasing the dimensionality by up to 10 more dimensions.

#### 3.2.2. Normalization and Standardization

The performance of regression, as well as the classification models, is seriously affected if the dataset columns contain values with different ranges. Mahfouz et al. [37] discussed how this problem leads to the performance of ML models deteriorating when various imbalanced scales of features have occurred in the dataset. Therefore, to deal with such problems, it is necessary to obtain the acceptable range for the negligible and dominant values. The two most popular techniques are min-max normalization and z-score standardization:

- Min–max normalization is used for transforming values of the dataset features into the range of [0, 1] according to the following equation:
  (1) $$X_{normalized}=\frac{X-X_{min\_value}}{X_{max\_value}-X_{min\_value}}$$
  where $X_{normalized}$ represents the normalized value, $X_{min\_value}$ and $X_{max\_value}$ are the border range of the desired interval, which is in this study [0, 1], and X is the original value that would be transformed within these ranges.
- Z-score standardization is used for rescaling dataset features so that they will have the properties of a standard normal distribution with mean μ=0 and standard deviation σ=1.
  (2) $$X_{normalized}=\frac{X-\mu }{\sigma }$$

Algorithm 2 shows the pseudocode of the one-hot encoding approach, minimum–maximum normalization and standardization techniques used in this study.
**Algorithm 2** Pseudocode of One-hot encoding, Min–Max Normalization and Z-score Standardization**Input**: dataset features F, class label C**Output**: Pre-processed dataset**MinMaxScaler** (D, F, i): $X_{normalized}$ = 0 max ← maximum value among all values of column i∈F in D min ← minimum value among all values of column i∈F in D $X_{normalized}\leftarrow \frac{X-X_{min\_value}}{X_{max\_value}-X_{min\_value}}$ // Equation (1) **Return**
$X_{normalized}$**Standardize**(D,F, i): $X_{normalized}$ = 0 μ ←mean value of column i∈F in D σ ←
standard deviation value of column i∈F in D $X_{normalized}\;\frac{X-\mu }{\sigma }$// Equation (2) **Return**
$X_{normalized}$**Begin**:D’← [ ] // Normalized/ Standardized datasetF← Hot-encoding dataset D**For each** item i∈F in (D)
**do**: D’← MinMaxScaler (D, F, i) // both min-max and z-score method is D’← Standardize (D, F, i) // executed separately**End For****End**

### 3.3. Feature Selection Techniques

As mentioned earlier, the N-BaIoT dataset consists of 115 features and 10 class labels, plus the “Benign” class that was added after encoding the target class. Passing this high dimensional vector into the ML model might cause a delay in the training and testing time of ML models. Consequently, any proposed attack detection system built with this issue usually consumes the processing resource very rapidly, which is not appropriate for the real-time systems. Therefore, the proposed approach first investigates how various filter-based feature selection techniques can be helpful for overcoming this issue. The impact of PCA, MI and the ANOVA f-test on the performance of ML models is explored. As presented in Section 4.1, the experimental results show that the MI filter-based technique yields the highest accuracy score when the binary dataset is used. An aggregated MI with different rank aggregation function is proposed and tested on the multi-class dataset (see Section 4.2). The idea behind the aggregated MI is described as follows:

Compute the mutual information score for each feature, $f_{i}$, in dataset D with respect to class type c∈ C. The features are then ranked based on the aggregator functions listed in Table 3. Only p% of features are retained and fed later to the classifiers listed in Table 3 and the overall performance is measured.

### 3.4. Classification Algorithms

In this work, two types of ML classifiers are used: (i) two ensemble-based classifiers: Random Forest (RF), XGBoost (XGB) and (ii) four standalone classifiers, namely: Gaussian Naïve Bayes (GNB), k-Nearest Neighbor (k-NN), Logistic Regression (LR) and Support Vector Machine (SVM). For tuning the hyper-parameters of these classifiers, the optimal values are estimated by using cross validation [38]. Typically, there are several hyper-parameter optimization techniques, among which the grid search, random search, Bayesian optimization and evolutionary-based optimization are commonly used techniques. In this work, the grid search was applied, and the results of the optimized process are shown in Table 4.

### 3.5. Model Evaluation Metrics

The most commonly used evaluation metrics were used to evaluate the performance of the ML classifiers, which are: Accuracy (Acc.), Precision (P), Recall (R) and F1 score. In addition to these metrics, the training time, prediction time and execution time of each classifier were computed. The full description of these metrics and how they are computed is presented in Table 5.

## 4. Results and Discussion

### 4.1. Preliminary Exploration Setup: Binary Dataset

**Figure 2:**
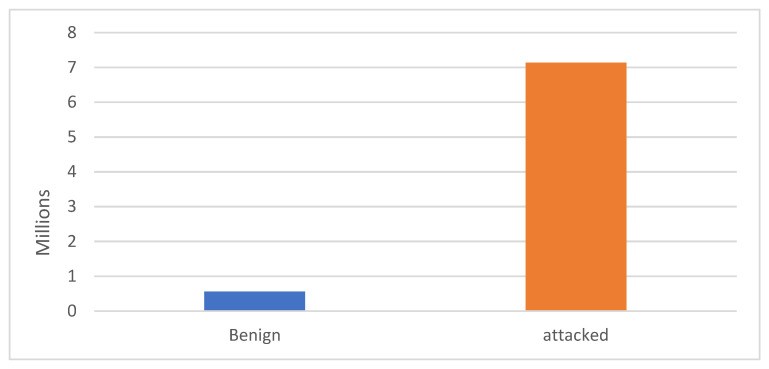
The distribution of attack and benign classes’ instances.

To conduct the experiment, the script was written in Python 3.7 using the Google Colab environment on the 64-bit Windows 10 operating system. The N-BaIoT dataset was organized in a way such that both “Bashlite” and “Mirai” classes were grouped together and formed one class, “attacked”. As shown in Figure 2, the number of the instances classified as “attacked” is much larger than the number of “benign” instances. Therefore, an under-sampling algorithm was applied on the class “attacked” to obtain a more balanced dataset. A balanced sample of the dataset was then used. Later, the obtained dataset was split into a training set and a testing set, using the *train_test_split* function found in the *sklearn* package, where 80% of data was used as the training dataset and the remaining data (20%) as the testing dataset. Table 6 presents the statistical outline of the balanced binary dataset used.

#### 4.1.1. Performance Exploration of Machine Learning Algorithm

Table 7 presents the performance of the used ML classifiers. The idea here is to investigate how the feature selection technique performs on the proposed binary dataset. Firstly, the ML-model is applied without using any FS technique. Then, different FS techniques are used. Table 7 shows the summarized performance of the ML classifiers in terms of accuracy.

#### 4.1.2. Discussion

**Figure 3:**
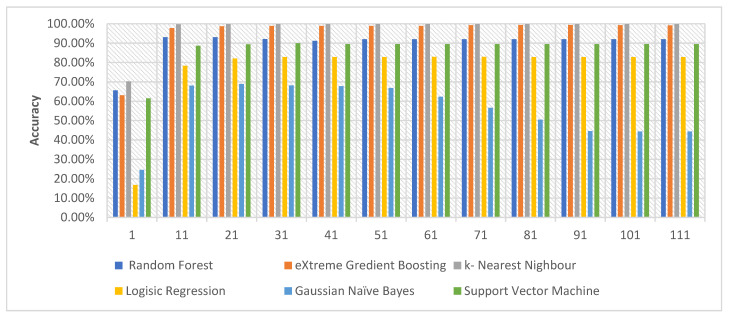
Accuracy of the ML model with respect to different PCA components.

Based on the results presented in Table 7, the following findings are observed and can be summarized as follows:

- k-NN and XGB classifiers yield the highest scores in terms of accuracy, which confirms the results reported in [20,21]. The k-NN exceeds all classifiers when all features are used.
- The performance of the classifiers is degraded when the PCA technique is used. The only exception is noted when SVM is used, when the number of components of PCA is 21, as shown in Figure 3 and Table A2.
- Most ML models benefit more when the MI feature selection technique is applied. The performance of ML classifiers in terms of accuracy exceeds the baseline, except LR, in which the performance decreased. As a result, the following section presents how MI can be beneficial for detecting attack types where the multi-class dataset is used. The proposed aggregated MI feature selection approach is highlighted.

### 4.2. N-BaIoT Dataset as a Multi-Class Dataset

To conduct the experiment fairly, the OvR strategy was applied. The reason behind this selection is its computational efficiency and interpretability. The OvR strategy represents each class by only one classifier, which allows knowledge to be gained about the class by inspecting its corresponding classifier.

**Figure 4:**
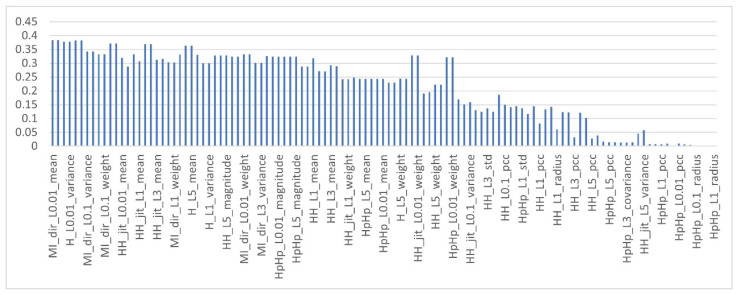
Mutual information of features with MAX aggregation function.

**Figure 5:**
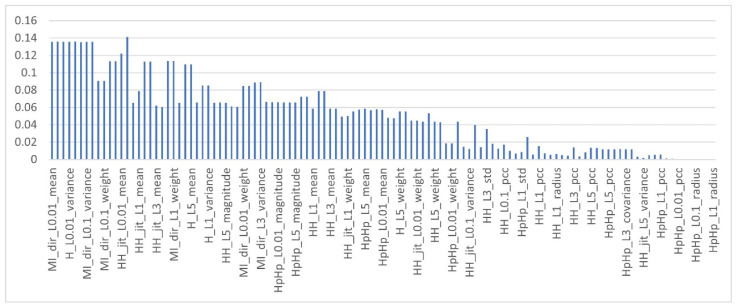
Mutual information of features with MIN aggregation function.

**Figure 6:**
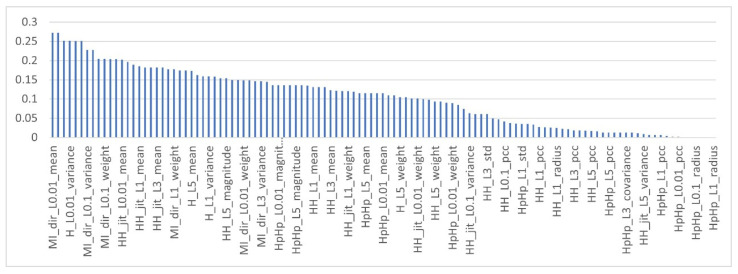
Sorted mutual information of Features with AVERAGE aggregation function.

To obtain the MI score of the features in the multi-class dataset, as mentioned earlier, each feature in the dataset is computed with respect to each class type, c∈ C, which means the target class is fixed using multiclass classification strategy (OvR) and the MI of the feature is computed with respect to this class type. As a result, each feature obtained 10 different MI scores. The features are then ranked based on the aggregator functions listed in Table 3. Figure 4, Figure 5 and Figure 6 show the mutual information scores of all features with respect to the MAX, MIN and AVERAGE aggregation functions.

As shown in the Figure 4, Figure 5 and Figure 6 above, each ranker search method ranks the attributes differently. The main issue with such methods, as with all filter-based FS methods, is that specifying the number of attributes that have to be retained is a subjective choice. In this work, only the top 10% of features were used that have the highest MI scores. Table 8 shows the names of the top 10% of features with respect to the aggregation functions.

#### Comparison of MI Feature Selection using Different Aggregation Functions

Based on these selected features, the performance of ML classifiers was now measured per each class type in terms of accuracy, precision, recall and F1score. In addition, the training time, prediction time and execution time were computed. Table 9 presents the accuracy of ML classifiers when features were selected based on different aggregation functions. Table 10, Table 11, Table 12, Table 13, Table 14 and Table 15 present the precision, recall and F1 score of these classifiers.

### 4.3. Discussion

This section meticulously analyzes the results listed in Table 9, Table 10, Table 11, Table 12, Table 13, Table 14 and Table 15. It also measures the performance of the employed classifiers in terms of time consumption. As shown in Table 9, the classifiers benefited differently when different aggregation operators were applied. The findings are summarized as follows:

- When the “MIN” and “AVERAGE” functions were used, the most of classifiers performed well and XGB, k-NN, GNB, LR and SVM achieved notable results compared to their results when the “MAX” operator was used. Among these methods, XGB obtained the best accuracy (99.19%).
- In most cases of the experiments, all classifiers showed good results when the “AVERAGE” operator was used as aggregation function, except RF and SVM.
- It is notable that RF benefited more only when the “MAX” operator was used as an aggregation function. The performance of RF was degraded a little.
- In terms of accuracy, XGB and k-NN classifiers achieved 99.19% and 98.28% respectively, which means that they are quite close. However, when their performances were measured in terms of time consumption, the preference tends to favor k-NN, since it consumes less time, as shown in Table 16.
- The prediction time is also a very important factor for employing an ML classifier for real-time applications. Thus, in the case that the ML classifier is used for preventing attacks on IoT devices in real-time and sensitive intrusion detection systems, the favor tends toward XGB.

Table 11 and Table 12 show the performance of the classifiers according to class types. The findings are summarized as follows:

- Among all attack types, the XGB and k-NN classifiers were capable of detecting the “*Mirai*” attack type perfectly.
- Among the “Bashlite” attack types that XGB was able to detect, the “TCP” and “UDP” attack types were poorly detected, whilst the k-NN classifier performed poorly with “TCP” and “UDP” attack types, and also with “COMBO” and “Junk” attack types.
- Interestingly, RF records the best performance with F1score of 100% for the “COMBO” attack type when the “AVERAGE” aggregation function was used. In addition, it achieved F1 score of 99.95% with the “Junk” type.

## 5. Conclusions

This paper has proposed an aggregated mutual information-based feature selection with machine learning methods for enhancing IoT botnet attack detection. The main phases of this method include data collection, data preparation, feature selection and classification using the N-BaIoT benchmark dataset. Each attack type was fed into the feature selection methods to obtain a set of reduced features. The set with reduced features was then used for training the ML classifiers using the OvR strategy. Finally, the ML model was evaluated and the overall performance was reported. The proposed method was applied for the binary (attack and benign) and multi-class (10 different attacks and benign) classification problems. The effect of PCA, MI and ANOVA f-test feature selection methods on the performance of ML models was investigated. Two ensemble-based classifiers: RF and XGB, and four individual classifiers: GNB, k-NN, LR and SVM methods with applying hyper-parameter methods were used in the conducted experiments. The evaluation of ML classifiers was performed by computing the accuracy, precision, recall and F1score. In addition to these metrics, the training time, prediction time and execution time of each classifier were computed. The experimental results showed that the MI filter-based technique yielded the highest accuracy score when the dataset of binary dataset was used. For the multi-class dataset, an aggregated MI with different rank aggregation functions was proposed and tested. The findings showed that, in terms of accuracy, XGB and k-NN classifiers achieved 99.19% and 98.28% respectively, while k-NN performed better for time consumption measure. Future works can apply the proposed method on different IoT botnet datasets. In addition, deep learning-based methods can be proposed and investigated to enhance IoT botnet attack detection.

## Figures and Tables

**Table 1:** Statistics of N-BaIoT dataset.

Feature Name		Number of Instances, %
IoT device types	Security cameras	1
	Webcam	1
	Smart baby monitor	1
	Thermostat	1
	Smart door-bell devices	2
General Features	Total number of Instances	6,273,053
	# of features in dataset	115
	Time windows	100 ms, 500 ms, 1.5 s, 10 s and 1 min
Distribution of data (2 classes)	# of “Benign” records	555,932 (7.23%)
	# of “attack” records	7,134,943 (92,77%)
Distribution of data (3 classes)	# of “Bengin” records	555,932 (7.23%)
	# of “Bashlite” records	2,838,272 (36,90%)
	# of “Mirai” records	4,296,671 (55,87%)

**Table 2:** The sampling of normal and attack classes in multi-class dataset.

Statistical Feature	Reference	Number of Records
“Benign”	$C^{1}$	555,932 (7.23%)
“Bashlite” attack type, % out of all instances	$C^{2}$	COMBO: 515,156 (6.698 %)
	$C^{3}$	Junk: 261,789 (3.403 %)
	$C^{4}$	Scan: 255,111 (3.317%)
	$C^{5}$	TCP: 859,850 (11.180%)
	$C^{6}$	UDP: 946,366 (12.305%)
“Mirai” attack type, % out of all instances	$C^{7}$	Ack: 865,646 (11.255%)
	$C^{8}$	Scan: 650,414 (8.457%)
	$C^{9}$	Syn: 790,227 (10.275%)
	$C^{10}$	UDP: 1,285,683 (16.717%)
	$C^{11}$	UDPplain: 704,701 (9.163%)

**Table 3:** Rank aggregation methods.

Aggregators	Formula	Description
Min ( )	$min\left\{R_{c_{1}}\left(f_{1\ldots n}\right),\;R_{c_{2}}\left(f_{1\ldots n}\right),\;.\;.\;.\;R_{c_{m}}\left(f_{1\ldots n}\right)\right\}$	Selects the *minimum* of the relevance scores produced when class type $c_{i}$ is used as a target class
Max ( )	$max\left\{R_{c_{1}}\left(f_{1\ldots n}\right),\;R_{c_{2}}\left(f_{1\ldots n}\right),\;.\;.\;.\;R_{c_{m}}\left(f_{1\ldots n}\right)\right\}$	Selects the *maximum* of the relevance scores produced when class type $c_{i}$ is used as a target class
Mean ( )	$mean\left(\left(\overset{m}{\underset{i=1}{\sum }}R_{c_{i}}\left(f_{1\ldots n}\right)\right)\times \frac{1}{m}\right)$	Selects the *mean* of the relevance scores produced when class type $c_{i}$ is used as a target class

**Table 4:** Classification Algorithms.

Classification Algorithms	Adjusted Parameters	Best Tuned Hyper-Parameter
RF	Criterion: [‘entropy’, ‘gini’] max_depth: [10–1200] + [None] max_features: [‘auto’, ‘sqrt’,’log2′, None] min_samples_leaf: [4–12] min_samples_split: [5–10] n_estimators’: [150–1200]	Criterion: ‘gini’, max_depth: 150, max_features: ‘auto’. min_samples_leaf: 4, min_samples_split: 7, n_estimators’: 150
XGB	n_estimators: [100–1200] max_depth: [1–11], learning_rate: [1 × 10^−3^, 1 × 10^−2^, 0.1, 0.5, 1.] subsample: [0.05–1.01] min_child_weight: [1–21]	n_estimators: 150, max_depth: 4, learning_rate: 1 × 10^−2^, subsample: 0.25. min_child_weight: 5
k-NN	leaf_size = [3–15], distance = [‘minkowski’, ‘Euclidian’, ‘Manhattan’] #neighbors = [3–45], p = 2, weights = ‘uniform’	leaf_size = 7, distance = ‘Manhattan’, #neighbors = 23, p = 2, weights = ‘uniform’
LR	C= [−4.0–4.0], intercept_scaling = 1, max_iter = [100–500], penalty = [‘l1′, ‘l2’], solver = [‘liblinear’, ‘lbfgs’], tol = 0.0001, verbose = 0	C= 1.0, intercept_scaling = 1, max_iter = 100, penalty = ‘l2’, solver = ‘lbfgs’, tol = 0.0001, verbose = 0
SVM	C = [0.1, 1, 10, 100, 1000] gamma = [1, 0.1, 0.01, 0.001, 0.0001] kernel = [‘rbf’, ‘kernel’]	C = 10 gamma = 0.001 kernel = ‘rbf’

**Table 5:** Evaluation metrics.

Measure Metric	Formula	Explanation
Accuracy (Acc.)	$\frac{TP+TN}{TP+TN+FP+FN}$	TP—Correctly classified instances as the right type of attack. TN—Correctly classified instances as benign. FN—Wrongly classified attack instances as benign. FP—Wrongly classified benign instances as an attack
Precision (P)	$\frac{TP}{TP+FP}$	
Recall (R)	$\frac{TP}{TP+FP}$	
F1 score	$\frac{\left(2\times Pre\times R\right)}{Pre+R}$	F1 score is the harmonic mean of precision and recall
Execution time $t_{e}$	$t_{e}=t_{1}+t_{p}$	$t_{1}$—Training time; $t_{p}$—Prediction time

**Table 6:** Number of samples for normal and attack classes in the training and testing dataset.

Class	Training Set	Testing Set
Benign	190,313	22,824
Attacked	191,927	72,736
Total Number of Records	382,240	95,560

**Table 7:** Exploration Investigation: Accuracy of ML models.

FS Technique	RF	XGB	k-NN	LR	GNB	SVM
Without	94.031%	99.382%	99.861%	82.631%	74.785%	89.189%
PCA	93.058%	99.290%	99.819%	82.053%	68.869%	89.928%
MI	94.391%	99.462%	99.903%	77.253%	84.819%	89.526%
ANOVA F-test	94.287%	99.294%	99.811%	80.157%	60.260%	88.645%

**Table 8:** Top 10% of Selected Features: The features are sorted in descending order with respect to MI score.

Aggregation Function	Feature Name
MAX	MI_dir_L0.01_mean H_L0.01_mean H_L0.1_mean MI_dir_L0.1_mean H_L0.01_variance MI_dir_L0.01_variance H_L1_mean MI_dir_L1_mean MI_dir_L3_mean H_L3_mean MI_dir_L5_mean H_L5_mean H_L0.1_variance MI_dir_L0.1_variance H_L0.01_weight
MIN	HH_jit_L0.1_mean H_L0.01_mean H_L0.1_mean H_L0.1_variance MI_dir_L0.01_mean MI_dir_L0.01_variance H_L0.01_variance MI_dir_L0.1_variance MI_dir_L0.1_mean HH_jit_L0.01_mean H_L1_weight MI_dir_L1_weight MI_dir_L1_mean H_L1_mean MI_dir_L3_mean
AVERAGE	MI_dir_L0.01_mean H_L0.01_mean MI_dir_L0.01_variance H_L0.01_variance H_L0.1_mean MI_dir_L0.1_mean MI_dir_L0.1_variance H_L0.1_variance H_L0.1_weight MI_dir_L0.1_weight H_L1_mean MI_dir_L1_mean HH_jit_L0.01_mean HH_jit_L0.1_mean HH_L0.01_magnitude

**Table 9:** Accuracy of classifiers with MI feature selection on the test dataset.

Classifier	Aggregation Function		
	MAX	MIN	AVERAGE
RF	0.9427	0.9414	0.9417
XGB	0.9386	0.9897	0.9919
k-NN	0.9305	0.9784	0.9827
LR	0.5896	0.6071	0.7513
GNB	0.7585	0.8464	0.8496
SVM	0.7612	0.8673	0.8201

**Table 10:** Performance analysis for N-BaIoT with RF and MI feature selection on the test dataset.

	Precision			Recall			F1score		
Class Name	MAX	MIN	AVE.	MAX	MIN	AVE.	MAX	MIN	AVE.
$C^{1}$	0.9994	0.9994	0.9978	1.0000	0.9998	0.9998	0.9997	0.9996	0.9988
$C^{2}$	1.0000	0.9995	1.0000	1.0000	1.0000	1.0000	1.0000	0.9998	1.0000
$C^{3}$	1.0000	1.0000	1.0000	0.9997	0.9992	0.9989	0.9999	0.9996	0.9995
$C^{4}$	1.0000	1.0000	0.9997	0.9997	1.0000	0.9994	0.9998	1.0000	0.9995
$C^{5}$	1.0000	0.8000	1.0000	0.0015	0.0014	0.0003	0.0029	0.0029	0.0007
$C^{6}$	0.5397	0.5390	0.5390	0.9997	0.9991	0.9985	0.7010	0.7002	0.7001
$C^{7}$	1.0000	0.9996	1.0000	1.0000	1.0000	1.0000	1.0000	0.9998	1.0000
$C^{8}$	1.0000	1.0000	1.0000	1.0000	0.9997	1.0000	1.0000	0.9998	1.0000
$C^{9}$	1.0000	1.0000	1.0000	1.0000	1.0000	1.0000	1.0000	1.0000	1.0000
$C^{10}$	1.0000	1.0000	1.0000	1.0000	1.0000	1.0000	1.0000	1.0000	1.0000
$C^{11}$	1.0000	1.0000	1.0000	1.0000	0.9995	1.0000	1.0000	0.9998	1.0000

**Table 11:** Performance analysis for N-BaIoT with XGB and MI feature selection on the test dataset.

	Precision			Recall			F1score		
Class Name	MAX	MIN	AVE.	MAX	MIN	AVE.	MAX	MIN	AVE.
$C^{1}$	0.9891	0.9727	0.9910	1.0000	0.9996	1.0000	0.9945	0.9859	0.9955
$C^{2}$	0.9988	0.9985	0.9995	0.9712	0.9689	0.9728	0.9848	0.9835	0.9859
$C^{3}$	0.9650	0.9724	0.9657	0.9934	0.9835	0.9971	0.9790	0.9779	0.9811
$C^{4}$	1.0000	1.0000	1.0000	0.9997	1.0000	0.9994	0.9998	1.0000	0.9997
$C^{5}$	1.0000	0.9234	0.9309	0.0015	1.0000	1.0000	0.0029	0.9602	0.9642
$C^{6}$	0.5397	0.9993	1.0000	0.9994	0.9281	0.9351	0.7009	0.9624	0.9665
$C^{7}$	1.0000	0.9998	1.0000	1.0000	0.9998	1.0000	1.0000	0.9998	1.0000
$C^{8}$	1.0000	1.0000	1.0000	1.0000	0.9997	1.0000	1.0000	0.9998	1.0000
$C^{9}$	1.0000	1.0000	1.0000	1.0000	1.0000	1.0000	1.0000	1.0000	1.0000
$C^{10}$	1.0000	1.0000	1.0000	1.0000	1.0000	1.0000	1.0000	1.0000	1.0000
$C^{11}$	1.0000	1.0000	1.0000	1.0000	0.9995	1.0000	1.0000	0.9998	1.0000

**Table 12:** Performance analysis for N-BaIoT with k-NN and MI feature selection on the test dataset.

	Precision			Recall			F1score		
Class Name	MAX	MIN	AVE.	MAX	MIN	AVE.	MAX	MIN	AVE.
$C^{1}$	0.9988	0.9982	0.9986	0.9998	0.9990	0.9998	0.9993	0.9986	0.9992
$C^{2}$	0.9963	0.9353	0.9431	0.9862	0.8959	0.9199	0.9912	0.9152	0.9313
$C^{3}$	0.9793	0.8471	0.8773	0.9940	0.9018	0.9114	0.9866	0.8736	0.8940
$C^{4}$	0.9988	0.9972	1.0000	0.9991	0.9988	0.9994	0.9989	0.9980	0.9997
$C^{5}$	0.4604	0.9993	0.9996	0.9985	0.9996	1.0000	0.6302	0.9995	0.9998
$C^{6}$	0.5000	0.9994	0.9997	0.0003	0.9991	0.9985	0.0006	0.9992	0.9991
$C^{7}$	1.0000	1.0000	1.0000	1.0000	0.9996	0.9996	1.0000	0.9998	0.9998
$C^{8}$	1.0000	1.0000	0.9997	0.9997	0.9994	1.0000	0.9998	0.9997	0.9998
$C^{9}$	1.0000	0.9997	1.0000	1.0000	0.9997	1.0000	1.0000	0.9997	1.0000
$C^{10}$	1.0000	1.0000	0.9998	1.0000	1.0000	1.0000	1.0000	1.0000	0.9999
$C^{11}$	0.9993	0.9998	1.0000	1.0000	0.9995	1.0000	0.9997	0.9997	1.0000

**Table 13:** Performance analysis for N-BaIoT with LR and MI feature selection on the test dataset.

	Precision			Recall			F1score		
Class Name	MAX	MIN	AVE.	MAX	MIN	AVE.	MAX	MIN	AVE.
$C^{1}$	0.2392	0.2747	0.3811	1.0000	0.9990	0.9998	0.3861	0.43091	0.5518
$C^{2}$	0.0000	0.4962	0.7715	0.0000	0.4478	0.5823	0.0000	0.47075	0.6637
$C^{3}$	0.0000	0.0000	0.0000	0.0000	0.0000	0.0000	0.0000	0.00000	0.0000
$C^{4}$	0.0000	0.9964	1.0000	0.0000	0.4268	0.4633	0.0000	0.59762	0.6332
$C^{5}$	0.0000	0.0000	1.0000	0.0000	0.0000	0.0004	0.0000	0.00000	0.0007
$C^{6}$	0.5397	0.5389	0.5390	0.9994	0.9991	0.9985	0.7009	0.70012	0.7000
$C^{7}$	1.0000	0.9992	1.0000	1.0000	0.9996	0.9994	1.0000	0.99939	0.9997
$C^{8}$	1.0000	0.9871	1.0000	0.7999	0.5693	0.9928	0.8889	0.72215	0.9964
$C^{9}$	0.8204	0.9990	1.0000	0.6615	0.1691	0.9015	0.7324	0.28920	0.9480
$C^{10}$	1.0000	1.0000	1.0000	0.7714	0.9079	0.9117	0.8710	0.95172	0.9538
$C^{11}$	1.0000	0.9998	1.0000	1.0000	0.9988	1.0000	1.0000	0.99931	1.0000

**Table 14:** Performance analysis for N-BaIoT with GNB and MI feature selection on the test dataset.

	Precision			Recall			F1score		
Class Name	MAX	MIN	AVE.	MAX	MIN	AVE.	MAX	MIN	AVE.
$C^{1}$	0.9722	0.9644	0.9687	1.0000	0.9996	0.9998	0.9859	0.9817	0.9840
$C^{2}$	0.5980	0.6103	0.6152	0.9934	0.9955	0.9973	0.7466	0.7567	0.7610
$C^{3}$	0.2727	0.4516	0.5833	0.0039	0.0036	0.0018	0.0078	0.0072	0.0037
$C^{4}$	0.9967	0.9920	1.0000	0.9243	0.9895	0.9911	0.9591	0.9907	0.9955
$C^{5}$	0.4603	0.4608	0.4609	0.9985	0.9986	0.9996	0.6301	0.6306	0.6309
$C^{6}$	0.0000	0.0000	0.0000	0.0000	0.0000	0.0000	0.0000	0.0000	0.0000
$C^{7}$	0.5519	1.0000	0.9943	1.0000	0.9996	0.9998	0.7112	0.9998	0.9971
$C^{8}$	1.0000	1.0000	1.0000	0.9972	0.9981	0.9991	0.9986	0.9991	0.9995
$C^{9}$	1.0000	1.0000	1.0000	0.9885	0.9857	0.9865	0.9942	0.9928	0.9932
$C^{10}$	1.0000	1.0000	1.0000	0.1190	0.9961	0.9927	0.2126	0.9981	0.9963
$C^{11}$	1.0000	1.0000	1.0000	1.0000	0.9958	0.9993	1.0000	0.9979	0.9997

**Table 15:** Performance analysis for N-BaIoT with SVM and MI feature selection on the test dataset.

	Precision			Recall			F1score		
Class Name	MAX	MIN	AVE.	MAX	MIN	AVE.	MAX	MIN	AVE.
$C^{1}$	0.3892	0.9038	0.5414	1.0000	0.9970	0.9996	0.5603	0.9481	0.7023
$C^{2}$	0.8252	0.6629	0.7140	0.6243	0.9608	0.6679	0.7108	0.7845	0.6902
$C^{3}$	0.2500	0.9718	1.0000	0.0003	0.1780	0.0005	0.0005	0.3009	0.0011
$C^{4}$	1.0000	0.9925	0.9994	0.9277	0.9781	0.9862	0.9625	0.9852	0.9928
$C^{5}$	1.0000	0.7500	0.5000	0.0011	0.0011	0.0004	0.0022	0.0022	0.0007
$C^{6}$.	0.5396	0.5387	0.5390	0.9994	0.9991	0.9985	0.7008	0.7000	0.7000
$C^{7}$	0.9998	1.0000	1.0000	1.0000	0.9996	0.9998	0.9999	0.9998	0999
$C^{8}$	1.0000	0.9985	1.0000	0.9997	0.9988	0.9991	0.9998	0.9986	0.9995
$C^{9}$	1.0000	0.9993	0.9995	0.6181	0.9970	1.0000	0.7640	0.9982	0.9998
$C^{10}$	1.0000	0.9992	1.0000	1.0000	1.0000	1.0000	1.0000	0.9996	1.0000
$C^{11}$	1.0000	1.0000	1.0000	1.0000	0.9995	1.0000	1.0000	0.9998	1.0000

**Table 16:** Classifiers’ Time Consumption with respect to Aggregation Functions.

Classifier	Training Time (s)			Prediction Time (s)			Execution Time (s)		
	MAX	MIN	AVERAGE	MAX	MIN	AVERAGE	MAX	MIN	AVERAGE
RF	181.343	192.288	178.371	2.998	3.059	3.06	184.495	195.497	181.578
XGB	239.309	229.42	227.967	0.670	0.758	0.722	240.138	230.357	228.852
K-nn	20.928	10.732	20.622	68.744	30.085	24.474	89.820	40.977	45.242
LR	18.285	24.574	23.204	0.034	0.04	0.037	18.516	24.815	23.445
GNB	0.874	0.95	0.916	0.210	0.223	0.202	1.232	1.333	1.267
SVM	3144.112	4235.9	3308.709	266.762	229.278	218.782	3411.02	4465.33	3527.637

## Data Availability

The dataset can be obtained from http://archive.ics.uci.edu/ml/datasets/detection_of_IoT_botnet_attacks_N_BaIoT (last accessed on: 6 December 2021; 23:00 GMT).

## Appendix A

**Table A1:** The full names of features in the N-BaIoT dataset.

F. No	Feature Name	F. No	Feature Name	F. No	Feature Name	F. No	Feature Name
$f_{1}^{MI}$	MI_dir_L5_weight	$f_{8}^{H}$	H_L1_mean	$f_{15}^{HH}$	HH_L1_weight	$f_{37}^{HH}$	HH_jit_L5_mean
$f_{2}^{MI}$	MI_dir_L5_mean	$f_{9}^{H}$	H_L1_variance	$f_{16}^{HH}$	HH_L1_mean	$f_{38}^{HH}$	HH_jit_L5_variance
$f_{3}^{MI}$	MI_dir_L5_variance	$f_{10}^{H}$	H_L0.1_weight	$f_{17}^{HH}$	HH_L1_std	$f_{39}^{HH}$	HH_jit_L3_weight
$f_{4}^{MI}$	MI_dir_L3_weight	$f_{11}^{H}$	H_L0.1_mean	$f_{18}^{HH}$	HH_L1_magnitude	$f_{40}^{HH}$	HH_jit_L3_mean
$f_{5}^{MI}$	MI_dir_L3_mean	$f_{12}^{H}$	H_L0.1_variance	$f_{19}^{HH}$	HH_L1_radius	$f_{41}^{HH}$	HH_jit_L3_variance
$f_{6}^{MI}$	MI_dir_L3_variance	$f_{13}^{H}$	H_L0.01_weight	$f_{20}^{HH}$	HH_L1_covariance	$f_{42}^{HH}$	HH_jit_L1_weight
$f_{7}^{MI}$	MI_dir_L1_weight	$f_{14}^{H}$	H_L0.01_mean	$f_{21}^{HH}$	HH_L1_pcc	$f_{43}^{HH}$	HH_jit_L1_mean
$f_{8}^{MI}$	MI_dir_L1_mean	$f_{15}^{H}$	H_L0.01_variance	$f_{22}^{HH}$	HH_L0.1_weight	$f_{44}^{HH}$	HH_jit_L1_variance
$f_{9}^{MI}$	MI_dir_L1_variance	$f_{1}^{HH}$	HH_L5_weight	$f_{23}^{HH}$	HH_L0.1_mean	$f_{45}^{HH}$	HH_jit_L0.1_weight
$f_{10}^{MI}$	MI_dir_L0.1_weight	$f_{2}^{HH}$	HH_L5_mean	$f_{24}^{HH}$	HH_L0.1_std	$f_{46}^{HH}$	HH_jit_L0.1_mean
$f_{11}^{MI}$	MI_dir_L0.1_mean	$f_{3}^{HH}$	HH_L5_std	$f_{25}^{HH}$	HH_L0.1_magnitude	$f_{47}^{HH}$	HH_jit_L0.1_variance
$f_{12}^{MI}$	MI_dir_L0.1_variance	$f_{4}^{HH}$	HH_L5_magnitude	$f_{26}^{HH}$	HH_L0.1_radius	$f_{48}^{HH}$	HH_jit_L0.01_weight
$f_{13}^{MI}$	MI_dir_L0.01_weight	$f_{5}^{HH}$	HH_L5_radius	$f_{27}^{HH}$	HH_L0.1_covariance	$f_{49}^{HH}$	HH_jit_L0.01_mean
$f_{14}^{MI}$	MI_dir_L0.01_mean	$f_{6}^{HH}$	HH_L5_covariance	$f_{28}^{HH}$	HH_L0.1_pcc	$f_{50}^{HH}$	HH_jit_L0.01_variance
$f_{15}^{MI}$	MI_dir_L0.01_variance	$f_{7}^{HH}$	HH_L5_pcc	$f_{29}^{HH}$	HH_L0.01_weight	$f_{1}^{Hp}$	HpHp_L5_weight
$f_{1}^{H}$	H_L5_weight	$f_{8}^{HH}$	HH_L3_weight	$f_{30}^{HH}$	HH_L0.01_mean	$f_{2}^{Hp}$	HpHp_L5_mean
$f_{2}^{H}$	H_L5_mean	$f_{9}^{HH}$	HH_L3_mean	$f_{31}^{HH}$	HH_L0.01_std	$f_{3}^{Hp}$	HpHp_L5_std
$f_{3}^{H}$	H_L5_variance	$f_{10}^{HH}$	HH_L3_std	$f_{32}^{HH}$	HH_L0.01_magnitude	$f_{4}^{Hp}$	HpHp_L5_magnitude
$f_{4}^{H}$	H_L3_weight	$f_{11}^{HH}$	HH_L3_magnitude	$f_{33}^{HH}$	HH_L0.01_radius	$f_{5}^{Hp}$	HpHp_L5_radius
$f_{5}^{H}$	H_L3_mean	$f_{12}^{HH}$	HH_L3_radius	$f_{34}^{HH}$	HH_L0.01_covariance	$f_{6}^{Hp}$	HpHp_L5_covariance
$f_{6}^{H}$	H_L3_variance	$f_{13}^{HH}$	HH_L3_covariance	$f_{35}^{HH}$	HH_L0.01_pcc	$f_{7}^{Hp}$	HpHp_L5_pcc
$f_{7}^{H}$	H_L1_weight	$f_{14}^{HH}$	HH_L3_pcc	$f_{36}^{HH}$	HH_jit_L5_weight	$f_{8}^{Hp}$	HpHp_L3_weight
$f_{9}^{Hp}$	HpHp_L3_magnitude	$f_{10}^{Hp}$	HpHp_L3_radius	$f_{11}^{Hp}$	HpHp_L3_covariance	$f_{12}^{Hp}$	HpHp_L3_pcc
$f_{13}^{Hp}$	HpHp_L1_weight	$f_{14}^{Hp}$	HpHp_L1_mean	$f_{15}^{Hp}$	HpHp_L1_std	$f_{16}^{Hp}$	HpHp_L1_magnitude
$f_{17}^{Hp}$	HpHp_L1_radius	$f_{18}^{Hp}$	HpHp_L1_covariance	$f_{19}^{Hp}$	HpHp_L1_pcc	$f_{20}^{Hp}$	HpHp_L0.1_weight
$f_{21}^{Hp}$	HpHp_L0.1_mean	$f_{22}^{Hp}$	HpHp_L0.1_std	$f_{23}^{Hp}$	HpHp_L0.1_magnitude	$f_{24}^{Hp}$	HpHp_L0.1_radius
$f_{25}^{Hp}$	HpHp_L0.1_covariance	$f_{26}^{Hp}$	HpHp_L0.1_pcc	$f_{27}^{Hp}$	HpHp_L0.01_weight	$f_{28}^{Hp}$	HpHp_L0.01_mean
$f_{29}^{Hp}$	HpHp_L0.01_std	$f_{30}^{Hp}$	HpHpL0.01_magnitude	$f_{31}^{Hp}$	HpHp_L0.01_radius	$f_{32}^{Hp}$	HpHp_L0.01_covariance
$f_{33}^{Hp}$	HpHp_L0.01_pcc	$f_{34}^{Hp}$	HpHp_L3_mean	$f_{35}^{Hp}$	HpHp_L3_std		

**Table A2:** Accuracies of Machine learning model with respect to different PCA components.

No. of Components	RF	XGB	k-NN	LR	GNB	SVM
1	65.605%	63.072%	70.231%	16.720%	24.553%	61.430%
11	93.011%	97.711%	99.765%	78.314%	68.009%	88.621%
21	93.058%	98.657%	99.802%	82.053%	68.871%	89.350%
31	92.066%	98.871%	99.819%	82.822%	68.179%	89.928%
41	91.145%	98.897%	99.817%	82.831%	67.753%	89.506%
51	92.055%	98.920%	99.817%	82.833%	66.803%	89.521%
61	92.043%	98.869%	99.817%	82.904%	62.286%	89.521%
71	92.051%	99.290%	99.817%	82.890%	56.603%	89.521%
81	92.049%	99.327%	99.817%	82.843%	50.457%	89.521%
91	92.043%	99.306%	99.817%	82.818%	44.553%	89.521%
101	92.055%	99.292%	99.817%	82.776%	44.333%	89.521%
111	92.051%	99.187%	99.817%	82.759%	44.333%	89.521%
